# Prenatal Maternal and Possible Transgenerational Epigenetic Effects on Milk Production

**DOI:** 10.1371/journal.pone.0098928

**Published:** 2014-06-05

**Authors:** Boyd Gudex, David Johnson, Kuljeet Singh

**Affiliations:** 1 Livestock Improvement Corporation, Hamilton, New Zealand; 2 University of New England, Armidale, Australia; 3 AgResearch, Ruakura Research Centre, Hamilton, New Zealand; CSIRO, Australia

## Abstract

This study investigated whether the prenatal maternal environment in dairy cattle influences the postnatal milking performance of the resulting daughters and grand-daughters. Linear mixed models were used to analyse whole season milk production from ∼46000 Jersey and ∼123000 Holstein Friesian cows in their 1^st^ and 2^nd^ lactations. Variation in the prenatal environment was associated with a small but significant (P<0.05) proportion of the total phenotypic variation (0.010 to 0.015) in all traits in Holstein Friesian cows and in the first lactation milk volume (0.011) and milk protein (0.011), and the second lactation milk fat (0.015) in the Jersey breed. This indicates that the prenatal environment does influence the adult performance of the subsequent daughter. Associations between daughter performance and dam and grand-dam traits indicative of their prenatal environment were also estimated. A one litre increase in the dam’s herd test milk volume was associated with a 7.5 litre increase in the daughters’ whole season milk yield and a 1% increase in either the dams’ herd test milk fat or protein percentage was associated with a reduction in daughter whole season milk volume (−49.6 and −45.0 litres for dam fat and protein, respectively). Similar results between the grand-dam herd test traits ansd the daughters’ whole season milk production were observed with a 1% increase in either grand-dam milk fat or protein percentage associated with a reduction in daughter whole season milk yield (−34.7 and −9.7 litres for fat and protein, respectively). This study revealed that the prenatal environment of the dam and the grand-dam can influence milk production in the subsequent daughters, though the effects are small. The similarity of the results between the dam daughter and the grand-dam daughter analyses suggests that the majority of the prenatal maternal effects are mediated by epigenetic mechanisms.

## Introduction

Due to the nutritional demands placed on a dairy cow by concurrent maintenance, growth, lactation and/or foetal development processes, energy requirements can exceed the amount of energy ingested at certain times of the year [1], [2], particularly for cows maintained on pasture with limited supplementation as found in New Zealand. The growth of the pasture is also subject to a variable climate. As a consequence, the quantity and quality of feed available to each cow can vary between locations or between years in the same location. The resulting energy deficit at this time forces the cow to either prioritise the demands and/or provide suboptimal levels of nutrition to each of these processes. Although the energy requirements of the developing foetus are low during early pregnancy compared to the other physiological processes, there is some evidence that foetal malnutrition can influence the resultant progeny’s performance later in life [1]–[5] and, potentially, also the performance of future generations [6], [7].

Epigenetics (changes in gene expression that occur in the absence of changes in the DNA sequence) have been identified as a mechanism by which the maternal environment influences the adult performance of the foetus. Until recently, epigenetic changes in mammals were only thought to last the lifetime of the affected organism and were reprogrammed in gametes, thus they are not passed on to further generations. However, recent research (for reviews see Gluckman [8], Daxinger and Whitelaw [9]) has revealed that some epigenetic marks can be passed on to the next generation where they continue to influence the phenotype. As epigenetic effects are not mediated by the DNA sequence variations that underpin additive genetic inheritance, but by how DNA is expressed, research in this field requires the additive genetic effects to be separated from the other prenatal maternal environment effects, such as the maternal genetic and maternal permanent environmental effects. Given sufficient data, the additive and prenatal maternal effects can be separated using typical quantitative genetics statistical models, and due to dairy calves being separated from their mothers soon after birth and hand reared, any maternal effects that are detected can be assumed to have occurred prenatally and not after birth [1], [3].

In order to detect any associations between the prenatal environment provided by the cow and her subsequent daughter or granddaughter’s performance, a measure of the prenatal environment is required. While it is not possible to directly measure a cow’s prenatal environment, traits that influence it (e.g. a cow’s nutritional or physiological status) can be measured and included in analyses. Previous studies have used age [2], [3], [10], disease presence, pregnancy and/or lactation [2] as measures of physiological status and body weight [11], [12], body condition score (BCS) [3], [10], [11], [12], dry matter intake [10], and/or milk production [1]–[3] as indicators of nutritional status in the dam. These studies found associations between the prenatal environment and reproduction [3], somatic cell score [1], milk yield [1], [2], [12], longevity [1], [2] and the sex of progeny born to the resulting daughters [11].

The objective of this study was to investigate whether the maternal environment influences the subsequent postnatal milking performance of the resulting daughter and grand-daughters under New Zealand conditions. The milking performance of the dam and grand-dam is used as a proxy measurement for the dam and grand-dams’ maternal environment as this is the most commonly recorded trait related to the maternal environment in New Zealand dairy cattle. The results of this study will contribute to future research to determine the ideal level of nutrition under which New Zealand dairy cows should be maintained during gestation for optimal female progeny performance.

## Materials and Methods

### Data

Milk production data from 46336 Jersey and 123268 Holstein Friesian cows (the ‘daughters’) and their dams (33760 Jersey and 90106 Holstein Friesian) and grand-dams (29184 Jersey and 76871 Holstein Friesian) were obtained from LIC’s sire proving scheme. The purpose of the sire proving scheme was to generate phenotypic data for the unbiased genetic evaluation of commercial sires and as such the collection of the data was not subject to ethics approval. The data is available for the purposes of reproducing these results from the author upon request. The data were edited to contain only single born/non embryo transfer daughters with known parentage, and dam and grand-dam daughter pairs that were born in the same location. These edits were to ensure that no bias due to differing maternal and daughter environments or abnormal reproduction occurred in the analysis. Separate datasets were created to allow the Jersey and Holstein Friesian breeds to be analysed separately and to qualify, each animal contained a minimum of 14/16^ths^ of the nominated breed. All daughters were born between 1986 and 2009. The dataset contained 7380 Jerseys and 17940 Holstein Friesians as both daughters and dams, and 2502 Jerseys and 6527 Holstein Friesians appeared as both daughters and grand dams.

The type of milk production data used varied between the generations. Yield deviations for milk volume, milk protein yield and milk fat yield were obtained for the whole of each of the daughters’ first two lactations (44140 and 33844 Jersey records and 117624 and 84029 Holstein Friesian records for lactation 1 and 2, respectively) and the results from a single herd test (single day milk production) obtained during the 1st trimester of the dam’s pregnancy that resulted in the daughter were obtained to be used as a proxy for the dam’s nutritional status/prenatal environment (Table 1). Likewise, a herd test result was obtained for the grand-dams but from the 1^st^ trimester of the pregnancy that produced the dam of the daughter (Table 1). The results from a single herd test were used as a proxy as it isolated milk production at a time where peak lactation and early foetal development occur concurrently. The use of a single herd test also allowed the proxy trait to be slightly differentiated from the response trait (whole season milk production). The daughter yield deviations were calculated within contemporary group, were based on a 270 day lactation and took account of milking frequency and the number and timing of herd tests (for more detail on the yield deviations see Johnson [13]). By pre-correcting the lactation traits, the statistical model was able to be simplified thus reducing computing requirements. Furthermore, the corrections were more accurate because they were derived from the whole database and not just those animals meeting the criteria of this study. Contemporary groups were defined by the daughter’s birth location and year, and groups containing less than 10 members were removed from the dataset leaving a total of 616 Jersey and 1487 Holstein Friesian contemporary groups. A pedigree file was also created that contained all of the known relationships between animals in the study, including ancestral relationships, and used in the analyses. These pedigree files contained ∼150000 Jerseys and ∼350000 Holstein Friesians.

**Table 1:** Number of records, average and the associated standard deviation for the dam and grand-dam herd test results.

Breed	Generation	Number of Records	Milk Trait	Units	Average (per herd test)	Standard Deviation
Jersey	Dam		Volume	Litres	15.0	3.7
		33760	Fat	%	5.4	0.8
			Protein	%	3.8	0.8
	Grand-Dam		Volume	Litres	15.7	3.9
		29184	Fat	%	5.3	0.8
			Protein	%	3.8	0.9
Holstein Friesian	Dam		Volume	Litres	20.5	5.3
		90106	Fat	%	4.3	0.7
			Protein	%	3.4	0.6
	Grand-Dam		Volume	Litres	20.6	5.1
		76871	Fat	%	4.4	0.7
			Protein	%	3.4	0.7

### Statistical Analysis

The estimation of variance components and the genetic merit of each individual (i.e. breeding values) in this study were carried out in a manner similar to that described by Berry [1] and Gudex [12]. The variance components/breeding values were estimated separately for each trait within parity using a linear model in ASREML [14] as follows:
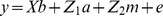
where Y is a vector of the phenotypic performance for each trait within each parity; b is a vector of fixed effects; a is a vector of animal; m is a vector of dam of animal; and e is a vector of residuals. The X and Z matrices are incidence matrices linking the vectors of fixed and random effects, respectively, to the vector of observations (Y). The fixed effects included contemporary group (as described in previous section) and the percentage nominated breed (Jersey or Holstein Friesian). A single herd test from either the dam or grand-dam was also added as a fixed effect to some analyses so that the daughter response to variation in the prenatal maternal environment of the dam or grand-dam could be estimated. The random effects of daughter, dam and residual were included to estimate the corresponding animal (additive genetic, v_a_), maternal (v_m_) and error (residual) variances (v_e_). From these variances, the phenotypic variance was calculated using v_p_ = v_a_+v_m_+v_e_, direct heritability using h^2^ = v_a_/v_p_ and maternal heritability using m^2^ = v_m_/v_p_.

## Results

The variance components for each of the traits analysed are summarized in Table 2. All direct additive genetic effects except lactation 1 protein yield in the Holstein Friesians were significantly different from zero (P<0.01), and the direct heritability estimates varied from 0.199 (lactation 2 fat yield in Jerseys) to 0.316 (lactation 1 milk volume in Holstein Friesians). The maternal genetic variance was only different (P<0.05) from zero for milk volume (in all lactations and breeds) and lactation 2 fat yield in the Jersey breed. The maternal genetic variance accounted for a relatively small proportion of each trait. The maternal heritability (m^2^) varied between 0.007 and 0.015 and was significant (P<0.05) for all traits apart from lactation 1 fat yield, and lactation 2 milk volume and protein yield in the Jersey breed.

**Table 2:** Variances of each of the components estimated and the resulting genetic parameters. Standard errors are given for each estimate.

				Variance				Maternal Heritability (m^2^)
Breed	Lactation	Milk Trait	Units	Additive Genetic	Maternal Genetic	Residual	Heritability (h^2^)	
Jersey	1	Volume	Litres	40160**	1457**	90681**	0.301±0.016**	0.011±0.005*
		Fat	Kilograms	68.3**	2.0^ns^	219.6**	0.237±0.015**	0.007±0.004^ns^
		Protein	Kilograms	34.0*	1.6^ns^	110.6^ns^	0.232±0.015**	0.011±0.005*
	2	Volume	Litres	51912**	1385**	116329**	0.304±0.018**	0.008±0.005^ns^
		Fat	Kilograms	72.4**	5.5**	291.2**	0.199±0.016**	0.015±0.007*
		Protein	Kilograms	45.2**	2.1^ns^	143.6*	0.238±0.017**	0.011±0.006^ns^
Holstein Friesian	1	Volume	Litres	65442**	2276**	142726**	0.316±0.011**	0.011±0.003**
		Fat	Kilograms	79.7**	4.9^ns^	235.7*	0.250±0.010**	0.015±0.004**
		Protein	Kilograms	42.7^ns^	1.9^ns^	141.7^ns^	0.233±0.010**	0.011±0.003**
	2	Volume	Litres	79014**	3678**	182014**	0.301±0.012**	0.014±0.004**
		Fat	Kilograms	91.3**	4.0^ns^	308.4**	0.227±0.011**	0.010±0.004*
		Protein	Kilograms	51.9**	2.8^ns^	176.6^ns^	0.227±0.011**	0.012±0.004**

**P<0.01;

*P<0.05;

ns = not significantly different from zero.

In Table 3, the associations between dam and the daughter lactation performance from the mixed model equations are summarized. Averaged across both breeds and both lactations, a 1% increase in either the dams’ herd test milk fat or protein percentage was associated with a reduction in daughter whole season milk volume (−49.6 and −45.0 litres for dam fat and protein, respectively). A one litre increase in the dam’s herd test milk volume was found to be associated with a 7.5 litre increase (on average) in the daughters’ whole season milk yield. Within these averages, the adverse effect upon milk production due to increases in the three dam herd test traits was greater in the Holstein Friesians (average −38.9 litres across the 3 dam traits) than in the Jersey breed (−19.1 litres) and smaller in lactation 1 (−25.6 litres) than in lactation 2 (−32.4 litres). The effect of the dam herd test traits upon daughter whole season milk fat and protein yield was less consistent than observed for daughter milk volume with only 17 out of 24 regression coefficients being significantly (P<0.05) different from zero. Furthermore, the sign of the response (positive or negative) was not consistent across breeds for their response to increases in dam protein percentage or milk volume. A 1% increase in dam milk fat percentage was associated with an increase in daughter whole season fat yield (1.57 kg) and a decrease in protein yield (−0.62 kg). The correlations between the daughter breeding values estimated from the base model (no dam or grand-dam traits included) and from models that included a dam prenatal environment proxy trait, varied between 0.86 and 0.93 with little variation observed in the correlations due to breed or lactation number.

**Table 3:** Regression coefficients of dam traits on daughter milking performance.

Breed	Prenatal Environment indicator trait	Lactation	Regression Coefficient		
			Volume (litres)	Fat (kg)	Protein (kg)
Jersey	Dam milk volume (litres)	1	7.95±0.60**	0.14±0.03**	0.16±0.02**
		2	9.18±0.79**	0.02±0.01*	0.18±0.03**
	Dam Fat %	1	−36.2±2.8**	0.84±0.13**	−0.41±0.09**
		2	−28.9±4.0**	0.77±0.17**	−0.83±0.12**
	Dam protein %	1	−29.8±3.8**	−0.01±0.17^ns^	−0.04±0.12^ns^
		2	−36.7±4.8**	−0.12±0.22^ns^	−0.08±0.16^ns^
Holstein Friesian	Dam milk volume (litres)	1	5.71±0.32**	−0.02±0.01*	0.07±0.10^ns^
		2	7.08±0.44**	−0.01±0.02^ns^	0.09±0.01**
	Dam Fat %	1	−53.4±2.4**	2.29±0.10**	−0.39±0.07**
		2	−79.7±3.2**	2.39±0.13**	−0.83±0.10**
	Dam protein %	1	−47.9±3.5**	0.87±0.14**	0.33±0.10**
		2	−65.4±4.6**	0.70±0.18**	0.16±0.14^ns^

Standard errors are given for each estimate.

**P<0.01;

*P<0.05;

ns = not significantly different from zero.

Table 4 summarizes the associations between grand-dam and the daughter lactation performance from the mixed models equations. Averaged across both breeds and lactations, a 1% increase in either the grand-dams’ herd test milk fat or protein percentage (from herd test) was associated with a reduction in daughter whole season milk volume (−34.7 and −9.7 litres for fat and protein, respectively). While the grand-dam’s herd test milk volume was found to be associated with an increase in daughter lactation 2 milk yield in Jerseys and both lactations in Holstein Friesians (average 4.2 litres), it also had a negative association with daughter milk yield (−13.7 litres) in Jersey lactation 1. Excluding the outlier (Jersey lactation 1), the average effect of the three grand-dam herd test traits was similar in the two breeds (Jerseys −15.3 litres and Holstein Friesians −14.5 litres) and smaller in lactation 1 (−9.9 litres) than in lactation 2 (−19.2 litres). The effect of the grand-dam’s herd test traits upon daughter whole season milk fat and protein yield was less consistent than observed on daughter milk volume with only 13 out of 24 regression coefficients being significantly different from zero, and the sign of the response (positive or negative) not being consistent across breeds. The correlations between the daughter breeding values estimated from the base model and from models that included a dam prenatal environment proxy trait varied between 0.85 and 0.89. The effect of the dam and grand-dam herd test traits on daughter performance was mostly consistent with only two cases where contrasting effects upon the daughter 1^st^ lactation milk volume were found (milk volume and protein percentage in the Jersey breed). All other daughter responses were consistent in sign, both positive or negative regardless of which parental generation herd test trait was included as a fixed effect in the model. In the majority of instances (10/12 for daughter milk yield, 7/12 for daughter milk fat and 9/12 for daughter milk protein), the daughter responses were greater when the dams’ herd test trait was included in the model (the average across all dam traits, breeds and lactations in daughters was −29.0 litres milk volume, 0.66 kg milk fat and −0.13 kg milk protein) compared with when the grand-dams’ herd test trait was included (−14.9 litres milk volume, 0.27 kg milk fat and −0.15 kg milk protein).

**Table 4:** Regression coefficients of grand-dam traits on daughter milking performance.

Breed	PrenatalEnvironment indicatortrait	Lactation	Regression Coefficient		
			Volume (litres)	Fat (kg)	Protein (kg)
Jersey	Grand-dam milk volume (litres)	1	−13.7±3.3**	0.09±0.03**	0.10±0.02**
		2	6.3±0.9**	0.14±0.04**	0.15±0.03**
	Grand-dam Fat %	1	−20.0±3.1**	0.17±0.14^ns^	−0.43±0.10**
		2	−52.0±3.6**	0.09±0.19^ns^	−0.64±0.14^ns^
	Grand-dam protein %	1	4.8±0.6**	−0.03±0.14^ns^	−0.16±0.11^ns^
		2	−17.1±4.1**	−0.19±0.18^ns^	−0.27±0.14^ns^
HolsteinFriesian	Grand-dam milk volume (litres)	1	2.52±0.36**	−0.03±0.01**	0.03±0.01**
		2	3.66±0.49**	−0.01±0.02^ns^	0.06±0.02**
	Grand-dam Fat %	1	−26.7±2.6**	0.99±0.10**	−0.25±0.79^ns^
		2	−40.0±3.6**	1.00±0.14**	−0.50±0.11**
	Grand-dam protein %	1	−10.2±2.9**	0.51±0.12**	0.14±0.09^ns^
		2	−16.2±3.8**	0.51±0.15**	0.02±0.11^ns^

Standard errors are given for each estimate.

**P<0.01;

ns = not significantly different from zero.

## Discussion

The results of this study confirm the findings of Berry *et al.* [1], Gonzalez-Recio *et al.* [2] and Gudex *et al.* [12], that maternal effects exist for milk production in dairy cattle. However, it is the association between the grand-dams’ milk production during pregnancy and the subsequent daughters whole season performance (grand-dam daughter pair analysis) that is most novel. While the associations between dam traits during pregnancy and subsequent daughter production (the dam daughter pair analysis) can be attributed to prenatal maternal effects, it is impossible to isolate epigenetic effects from the direct effect of the prenatal environment on embryo development (e.g. malnutrition of the embryo) [7], [8]. By analysing individuals that are 2 generations apart (the grand-dam daughter pairs), the grand-dams’ prenatal environment cannot impair the daughters development unless by transgenerational epigenetic mechanisms. These transgenerational epigenetic effects occur either by epigenetic marks being inherited from one generation to another or the prenatal environment influencing the methylation of DNA contained with the eggs (daughter generation) forming inside the developing embryo (dam generation) [2]. Epigenetic studies in the paternal line do not require three generations to study as sperm are produced after sexual maturity and thus they are not exposed to the prenatal environment [9]. Previous studies have reported transgenerational epigenetic inheritance in humans (e.g. the Dutch Famine Birth Cohort [15] and Overkalix Cohort [16] studies) and rodents, but not in cattle.

The presence of transgenerational epigenetic effects has numerous potential implications for the genetic evaluation of livestock as it may explain some of the missing causality and heritability observed in genomic studies of complex traits and could increase the accuracy of current genetic evaluations by accounting for some of the previously unknown variation [17]. The presence of small but significant maternal heritability for most of the daughter milk traits also implies that the statistical models used in the genetic evaluation of dairy cattle could include maternal genetic effects to improve variance partitioning and breeding value estimation, even though dairy calves are hand raised. Understanding of transgenerational epigenetics may also allow the prevention and/or treatment of diseases and other health issues by managing the environment during development and/or by new drug treatments that modify the molecular causes of epigenetic effects on genomic regions associated with development and disease. Selection of livestock that are less susceptible to the molecular causes of epigenetics and thus the resulting phenotypic changes may also be possible [17].

The association between the dam’s milk production during pregnancy and the subsequent daughter’s lactation performance (dam daughter pair analysis) detected in this study were similar to those reported by Berry *et al.* [1] and Gonzalez-Recio *et al.* [2] but are in contrast with those of Banos *et al.* [3] who found no association. Given that the associations observed in most of the dam daughter analyses were similar in magnitude to those observed in the corresponding grand-dam daughter analyses, it can be assumed that the majority of the variation observed in the dam-daughter analysis is also epigenetic and that the direct effect of the prenatal environment only has a (relatively) small effect. The results from the dam daughter analyses also support the ‘foetal origin hypothesis’ proposed by Barker *et al.* [18] where the environment *in utero* may lead to permanent effects in the subsequent generation, possibly by predisposing the individual to a more conservative metabolism. For example, as a response to poor prenatal nutrition, an individual may store more body tissue in adult life in anticipation of future potential adverse conditions in preference to milking to its full genetic potential. The association between the dam’s milk production and the daughter’s lactation performance may also explain the negative correlation between the additive genetic and maternal genetic effects observed in studies that investigate genetic parameters in livestock [1], [3]. With the energy required for high levels of milk production, as well as the demands for maintenance, growth and/or foetal development processes, the development and consequent adult performance of the embryo may be compromised leading to the negative association between the additive genetic and maternal genetic effects [1]–[3].

In this study and previous studies by Banos *et al.* [3], Berry *et al.* [1] and Gonzalez-Recio *et al.* [2], the milking performance of the dam and grand-dam is used as a proxy measurement for the dam and grand-dams’ prenatal maternal environment because this is the most commonly recorded trait that is related to the prenatal environment. With the proxy trait for the prenatal environment being so highly related to the response variable (daughter whole season lactation performance), the statistical model used to analyse this data had to account for the genetic relationships between the two traits. Gonzalez-Recio *et al.* [2] used a two stage Bayesian approach that firstly adjusted for the additive genetic relationships and fixed effects, then modelled the residuals with their prenatal maternal environment proxies (milk production, age, presence of lactation, pregnancy or disease). Maternal genetic effects were not fitted. In our study and those of Berry *et al.* [1], Banos *et al.* [3] and Gudex *et al.* [12], a linear mixed model that included the additive genetic and maternal genetic components as random effects and the prenatal environment proxy as a fixed effect was used. These models all have large data requirements for accuracy and, unfortunately due to the nature of this type of study, the amount of data available for analysis was reduced by half with every generation due to the birth of bull calves. The estimation of maternal effects also ideally requires multiple progeny per cow so that the per cow estimations are formed from more than a single progeny’s performance. As a response to these requirements, Banos *et al.* [3] only included dams with multiple daughters in production. The disadvantage of this approach is that it introduces year/dam age/dam parity effects which must be accounted for in the model and it was not used in this study or by Gudex *et al.* [12].

Other traits have been used as proxies for the prenatal maternal environment and include cow age [2], [3], [10], presence of disease/pregnancy/lactation [2], body weight [11], [12], BCS [3], [10], [11], [12] and dry matter intake [10]. The main limitation with using these traits in a study involving dairy cattle is that they are either not recorded in sufficient numbers and/or are not recorded during early pregnancy. For example, from the same dataset as used in this study (all with milk production recorded), only 5.9% of the cows had their BCS and live weight recorded during early pregnancy, thus greatly reducing the amount of available data [12]. The accuracy of both BCS and live weight as proxies for nutritional status is adversely affected by variation between individual cows in their ability to mobilise their body reserves. In an ideal situation, subjecting pregnant cows to contrasting levels of nutrition would provide a direct and quantifiable nutritional effect which removes any inaccuracies associated with the use of proxies. However, lactation would be adversely affected in the low nutrition group and, as a consequence, this approach is impractical in commercial settings similar to those from which these records were obtained.

The comparison of the daughters’ 1^st^ and 2^nd^ lactation results revealed that the associations between the dam/grand-dam herd test traits and daughter whole season lactation performance were on average smaller in the daughter’s 1^st^ lactation than in their 2^nd^ lactation. The reduction in association with parity could be due to wide variety of reasons. These reasons include that cows are still growing during their 1^st^ lactation, whereas in lactation 2 they are closer to mature size [3]. Older animals also produce more milk (and thus have more variation and potentially have accumulated more unfavourable mutations in the germ line and/or a larger amount of epigenetic marks that can be transmitted to the embryo [2]. The larger associations between the prenatal environment and the daughters’ lactation performance in the Holstein Friesians when compared with the Jerseys in this study could also be due to the greater milk volume produced by the Holstein Friesians. This also follows the observations of Gonzalez-Recio *et al.* [2] who noted that the negative effects of the concurrence of gestation and lactation on foetal programming was more noticeable in cows that produced more milk.

## Conclusions

This study indicates that small maternal and epigenetic effects (estimated separately) on lactation performance exist in New Zealand dairy cattle. The design of such studies are challenging as an accurate measure of the prenatal environment is required. Furthermore, any data from dams or grand-dams giving birth to a son is unusable and thus large datasets are required to distinguish between genetic and epigenetic mechanisms. However, this trial confirms the unique suitability of dairy cattle for maternal line transgenerational epigenetic research, firstly due to the hand rearing of calves eliminating postnatal maternal effects, and secondly, the availability of large multi-generation datasets. This research has implications for how the genetic evaluation of dairy cattle should be carried out and in the future may provide novel cow management and treatment of disease options.
